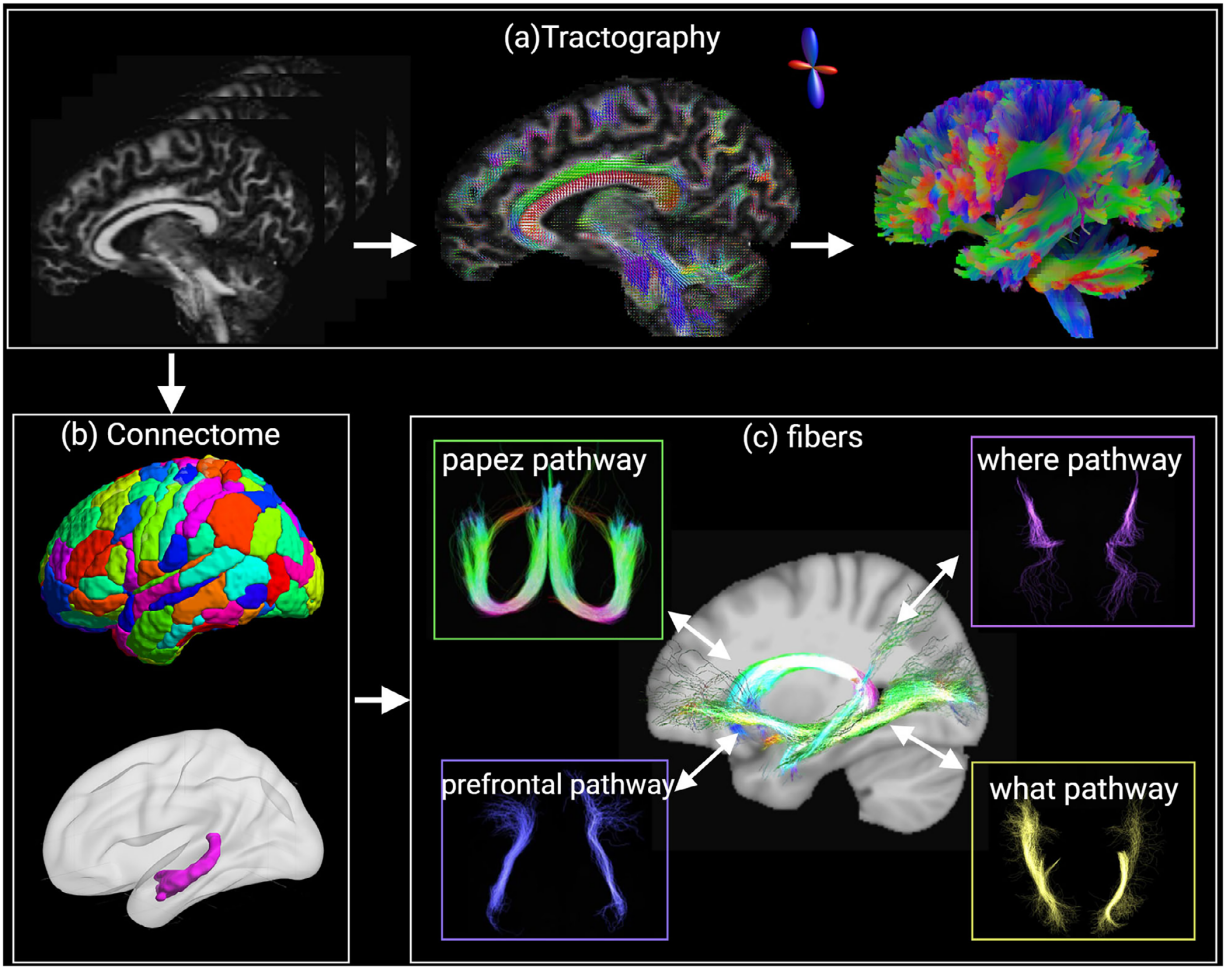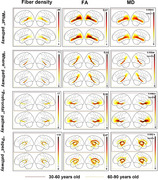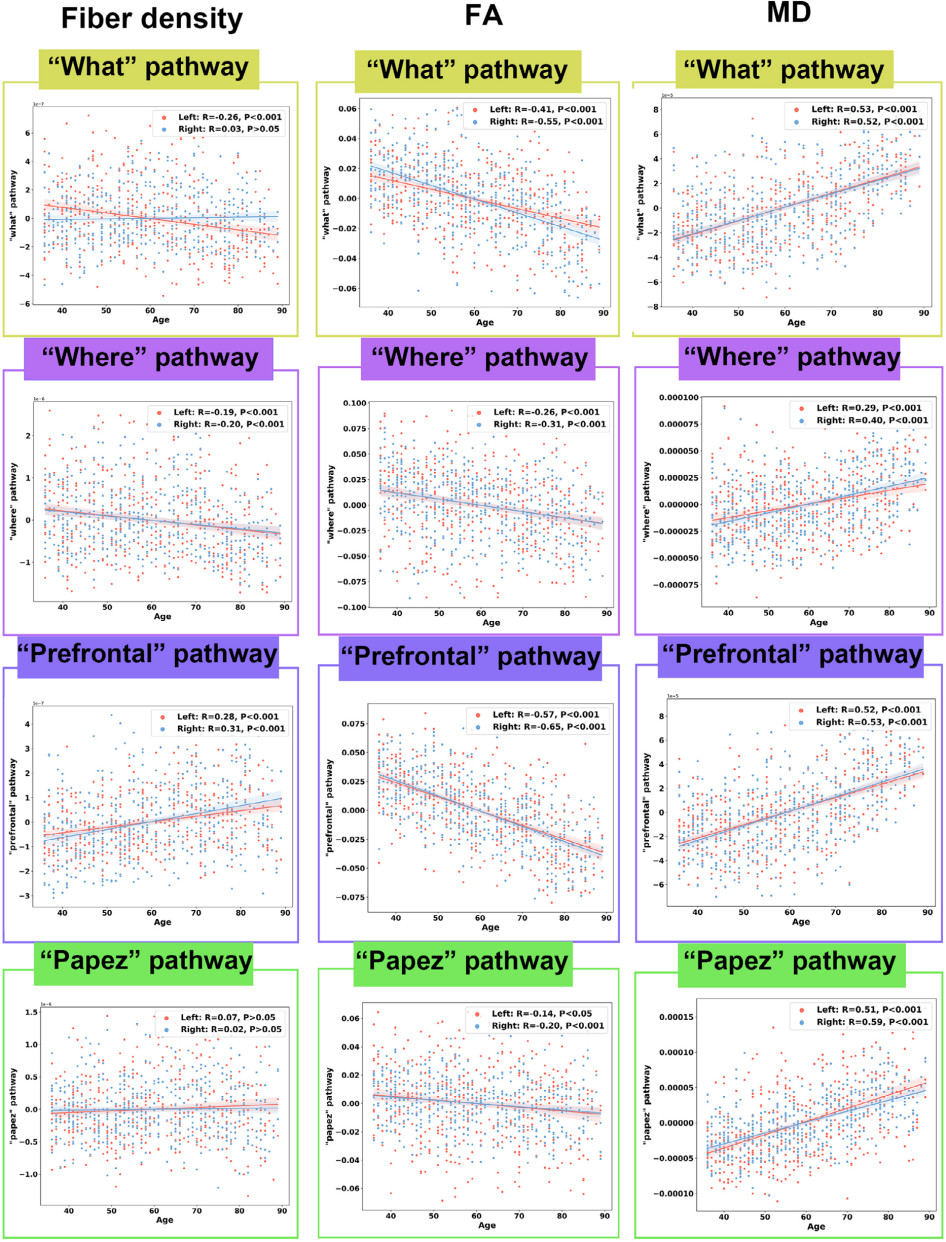# Imaging age‐related hippocampal connectivity changes across the adult lifespan in a large‐scale dataset

**DOI:** 10.1002/alz70856_102305

**Published:** 2025-12-25

**Authors:** Huize Pang, Chenyang Li, Zifei Liang, Jiaqi Wen, Zhe Sun, Henry Rusinek, Jiangyang Zhang, Yulin Ge

**Affiliations:** ^1^ NYU Grossman School of Medicine, New York, NY, USA; ^2^ New York University Grossman School of Medicine, New York, NY, USA; ^3^ NYU Alzheimer's Disease Research Center, New York, NY, USA; ^4^ Vilcek Institute of Biomedical Science, New York University School of Medicine, New York, NY, USA

## Abstract

**Background:**

The hippocampal networks link the hippocampus and several cortical areas, supporting memory, attention, and learning. Key pathways include the “where” pathway (medial hippocampus to dorsal visual cortex), “what” pathway (lateral hippocampus to ventral visual cortex), “prefrontal” pathway (hippocampus to the prefrontal cortex), and “papez” pathway (hippocampus and limbic regions). Although age‐related hippocampal volume decline is well‐documented, changes in its connectivity with aging remain unclear. This study investigates age‐related hippocampal connectivity changes using advanced diffusion MRI in a large‐scale dataset.

**Method:**

Tractography data were collected from 480 participants aged 36–90 years from HCP‐Aging dataset. Whole‐brain tractography was generated from diffusion MRI data, preprocessed with the DESIGNER pipeline. Tractography was reconstructed, with terminations constrained to the gray matter‐white matter interfaces. This process utilized fiber orientation distributions (FOD) from the MSMT‐CSD model, anatomically constrained tractography with iFOD2, and the SIFT methods. Whole‐brain connectome was generated based on brain parcellations from co‐registered structural MRI using MRcloud. Main hippocampal pathways were then extracted from the whole‐brain connectome using predefined inclusion and exclusion regions (Figure 1). Hippocampal pathways were characterized using fiber density (FD), fractional anisotropy (FA) and mean diffusivity (MD). Spearman correlation analysis was performed between hippocampal pathways characteristics and age, with Bonferroni correction.

**Result:**

Fiber density decreased in the left “what” pathway and bilateral “where” pathways and increases in the “prefrontal” pathway with age(*p* <0.001). FA values across all four pathways exhibited significant negative correlations with age (*R* from ‐0.14 to ‐0.65, *p* <0.05), whereas MD values showed significant positive correlations with age (*R* from 0.29 to 0.59, *p* <0.001) (Figures 2 and 3).

**Conclusion:**

This study revealed age‐related degeneration in key hippocampal pathways, evidenced by reduced FA, supporting previous volumetric and perfusion findings of hippocampus. Notably, the increased fiber density pathways connecting the hippocampus to the prefrontal cortex may suggest a compensatory mechanism, but this requires further validation. These findings may offer insights into early age‐related axonal and microstructural alterations underlying macroscale hippocampal changes in later life.

**Figure 1**. Workflow of key hippocampal pathways tractography.

**Figure 2**. Group‐level representation of hippocampal pathways in young and old groups.

**Figure 3**. Correlation analysis between hippocampal pathway measurements and age.